# Understanding drivers of private-sector compliance to large-scale food fortification: A case study on edible oil value chains in Bangladesh

**DOI:** 10.1016/j.foodpol.2021.102127

**Published:** 2021-10

**Authors:** Ayako Ebata, Jodie Thorpe, Ainee Islam, Sabiha Sultana, Mduduzi N.N. Mbuya

**Affiliations:** a Institute of Development Studies, Library Road, Brighton BN1 9RE, United Kingdom; b Innovision Consulting, House 26, Road 6, Block J, Pragati Sharani, Baridhara, Dhaka, Bangladesh; c Global Alliance for Improved Nutrition (GAIN) Bangladesh, Index Development Limited, House 20, Road 99, Dhaka, Bangladesh; d Global Alliance for Improved Nutrition (GAIN), 1701 Rhode Island Ave NW, Washington, DC 20036, USA

**Keywords:** Value chains, Nutrition, Public Health, Food fortification, Asia, Bangladesh

## Abstract

Micronutrient deficiency is a pertinent global challenge that affects billions of people and has deleterious health effects. Large-scale food fortification (LSFF) is a cost- effective way to tackle micronutrient deficiency and improve health outcomes, particularly in low- and middle-income countries (LMICs). However, the success of LSFF in LMICs is often hampered by limited compliance with fortification mandates by the private sector, who supply fortified foods. In this paper, we use a case study of the edible oil produced in Bangladesh to analyze the factors facilitating and impeding this compliance by for-profit actors. We identified four bottlenecks that disincentivize private sector actors’ decision to comply. First, fortified and non-fortified products co-exist in the market, disincentivizing producers to invest in fortification. Second, the lack of traceability reduces the risk for large-scale producers’ non-compliance with the regulation. Third, small-scale producers face economic pressures that prevent them from adequately fortifying oil products. Lastly, law enforcement is currently inconsistent, allowing the supply of under-fortified oil in the market. Given the evidence, we recommend to strengthen the control of bulk item fortification through more frequent and rigorous surveillance at the production level. This will ensure that resource constrained consumers who also have the greatest potential to benefit from added nutrients, remain able to access affordable and nutrient-enriched food.

## Highlights

• Co-existence of non-fortified products disincentivizes producers to fortify.
• Limited product traceability disincentivizes producers to comply with the policy.
• Economic pressure prevents small-scale producers from adequately fortifying.
• Political tension leads to inconsistent law enforcement, and limited compliance.
• Enforcement should focus on the origin of bulk items by large-scale producers.

## 1 Introduction

Micronutrient deficiency is a pertinent global challenge: at least 2 billion people are estimated to be deficient in at least one of the essential micronutrients needed for growth, development, and survival (Bailey et al., 2015). Among key micronutrients, vitamin A deficiency is widespread and can increase the risk of morbidity and mortality (Bailey et al., 2015). In Bangladesh, for instance, one in every five children of school age was estimated to be vitamin A deficient according to the 2011/2012 National Micronutrient Survey (icddr’b et al., 2013). In tackling micronutrient deficiency, large-scale food fortification (LSFF hereafter) is seen as a cost-effective means to improve nutritional and functional outcomes (Method and Tulchinsky, 2015). A recent systematic review estimates that LSFF with vitamin A could protect around 3 million children in low- and middle-income countries (LMICs hereafter) per year from vitamin A deficiency (Keats et al., 2019). Fortifying widely consumed food items – e.g., staples and condiments – can significantly increase micronutrient intake by poor and marginalized people who lack access to these critical nutrients (Raghavan et al., 2019).

However, LSFF in LMICs is often implemented and delivered sub-optimally, thereby limiting its potential for impact. For instance, Aaron et al. (2017) shows that compliance to fortification of chosen food items was less than half for 13 out of 18 LSFF programs across LMICs. Strikingly, the coverage of LSFF was lower among poorer and marginalized segments of the population (Aaron et al., 2017). Often, food vehicles selected for LSFF are widely consumed by the entire population (Mkambula et al., 2020). However, the fortification of these food items is constrained by challenges faced by value chain actors who are key players in LSFF in LMICs. Across LMICs, the private sector, alongside consumers, bear the majority of the cost of LSFF programs as governments and donors contribute 10% of all funds needed to make LSFF sustainable (Darnton-Hill et al., 2017). The large body of literature on the effectiveness of LSFF in LMICs indicates that for-profit private sector actors report several key economic barriers to complying with fortification regulations including high cost of premix (the powdery blend of vitamins and minerals used in fortification), competition with non-fortifying producers, and unclear regulatory requirements (Luthringer et al., 2015).

While private sector actors have the potential to contribute to nutritional outcomes, the conditions required for them to play a substantial role in providing nutrient-rich food to poor and marginalized people in LMICs are not well understood (Gillespie et al., 2013). To improve our understanding with respect to this gap, this article uses the experience of LSFF of edible oil in Bangladesh as an exploratory case study. We aim to generate new insights into the factors influencing the private sector’s contribution to nutritional outcomes, by seeking to understand the drivers of private sector non-compliance that have limited the success of Bangladesh’s edible oil fortification program.

In Bangladesh, edible oil was selected as a food vehicle for LSFF as it is widely consumed by the population (Kar, 2018). The Government initiated its effort to fortify edible oil with vitamin A in November 2013 (Vitamin A Enrichment in Edible Oil Act (Act No. 65 of 2013)) and officially mandated it under the “Oil Fortification Rules 2015” in November 2015 (The Fortification of Edible Oil with Vitamin 'A' Rules, 2015). The law stipulates the fortification of all edible oils with vitamin A and the levels of vitamin A that should be ensured and applied to locally produced, refined or imported products (Jungjohann et al., 2021). However, only 29% of evaluated edible oil products was fortified above the minimum standard (i.e., 15 mg/liter) (GAIN and icddr’b, 2017). Non– and under-compliance was found significantly more prominent in bulk, i.e., untraceable, oil products (7% compliance) than bottled oil (69% compliance) (GAIN and icddr’b, 2017).

In response to these findings, the Government of Bangladesh is currently considering stronger legislation regarding bulk oil transportation in drum and appropriate labelling to ensure traceability to strengthen surveillance (Government of Bangladesh, 2020). Banning bulk food items due to concerns of supplying under- or non-fortified food is one measure which has been considered in Indonesia (Jus’at and Soekirman, 2019) and tried out in Pakistan with limited success (Randall and Anjum, 2014). In addition, banning bulk oil trade might significantly affect access to edible oil by poor and marginalized populations. Against this backdrop, our study identifies ways to improve private sector compliance with fortification regulations without jeopardizing the access to fortified commodities based on an in-depth qualitative study in Bangladesh.

The rest of the article is organized as follows. In Section 2, we discuss the context of edible oil industry in Bangladesh and review literature that discusses the challenges faced by for-profit value chain actors in contributing to successful LSFF programs in LMICs. In Section 3, we explain our data collection strategies and methods of analysis before presenting our results in Section 4. In Section 5, we discuss our results to recommend ways to improve food fortification in LMICs before concluding in Section 6.

## 2 Backgrounds

### 2.1 The edible oil industry in Bangladesh

**Fig. 1:**
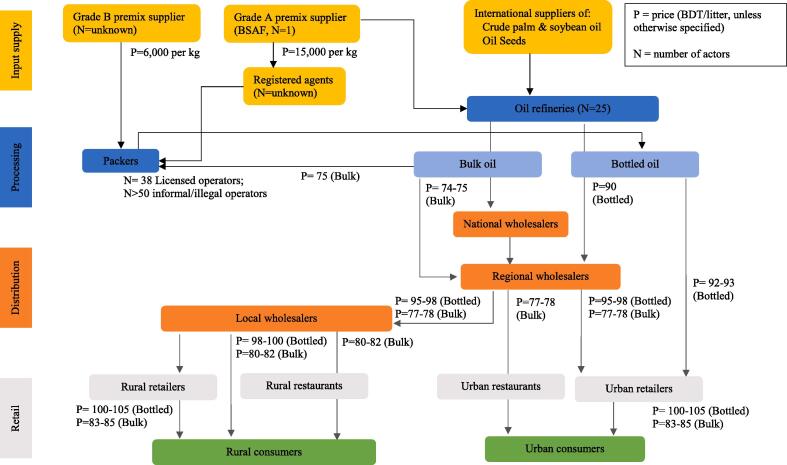
Edible oil value chains in Bangladesh.

We divide the edible oil industry in Bangladesh in two value chains (Fig. 1): 1) bulk edible oil, consisting of soybean, palm and super palm oil and representing approximately 65% of the market share in the country and 2) bottled edible oil which take up the remaining 35% (GAIN and icddr’b, 2017)^1^. Bottled oil includes 39% soybean oil, 20% rice bran oil, 19% sunflower oil, 18% palm oil, 3% vegetable oil, 1% canola oil and 1% super palm oil. This study focuses on bulk and bottled palm oil, soybean oil and super palm oil as the major sources of edible oil refined and processed in Bangladesh.

80 to 90% of edible oil consumed in Bangladesh is refined and processed domestically after crude oil is imported (OPM et al, 2020a)^2^. The edible oil sector is concentrated at refinery level with a total of 25 refinery groups, all of whom are large-scale producers. These refineries manufacture and sell both bulk and bottled oil. However, there are a larger number of oil packers, including a total of 38 registered packers and more than 50 unregistered packers (estimated). These small- and medium-scale producers procure refined bulk oil from refineries, which they bottle and distribute^3^.

Currently, only 69% of bottled oil and 7% of bulk oil is fortified above the minimum standard^4^ set by the Government of Bangladesh (GAIN and icddr’b, 2017). While refineries are responsible for fortification of both bulk and bottled oil, fortification of bulk oil is unsatisfactory (GAIN and icddr’b, 2017). As a result, regulations require packers as well as refineries to fortify edible oil with vitamin A premix. Two types of premix were reported to be available in Bangladesh: a Grade A quality premix, produced by BASF and sold at Bangladeshi Taka (BDT hereafter) 15,000/kg, and a Grade B quality premix, sold at BDT 6000/kg^5^. Wholesalers and retailers are not responsible for fortifying oil or checking the fortification of oil they handle.

Bulk oil is transported directly from refineries to national-level wholesalers in oil drums provided by wholesalers with little to no traceability. Bulk oil is purchased by institutional customers such as bakeries and restaurants and in loose form by individual consumers. Retailers acquire bulk oil from regional wholesale markets in cities such as Dhaka and Chittagong and bottled oil from sales representatives of different brands. Bottled oil is clearly labeled with the name of brands (i.e., associated with refineries or packers). Generally, retailers stock both bottled and bulk oil even in urban middle-income markets where the demand for bottled oil is higher than in other income groups. On average, bottled oil is significantly more expensive at 143 BDT than bulk oil at 85 BDT (GAIN and icddr’b, 2017).

### 2.2 Literature review: Factors influencing fortification performance

According to Luthringer et al. (2015), private sector actors report their greatest barrier to compliance with LSFF as being the cost of and access to premix. In Bangladesh, although the Ministry of Industries (MoInd hereafter) had initially procured vitamin A premix for oil refineries, this support has ceased when edible oil fortification became mandated in 2017 (MDF Training and Consultancy BV, 2017). When premix is imported from abroad, it is subject to import duties, value-added tax (VAT), and currency exchange rates (Garrett et al., 2016, Lalani et al., 2019), resulting in increasing and fluctuating price. For large-scale enterprises, the cost of premix is merely 0.1–0.2% of the total cost (Chaudhry, 2018) and they are able to compensate for this cost increase through gaining efficiency in other activities (Lalani et al., 2019). On the contrary, this proves challenging for small- and medium-scale enterprises who require small quantity of premix and undertake a limited number of activities through which they could absorb the increased cost of fortification (Garrett et al., 2016). In addition to premix, there is a cost associated with adapting to and introducing new processing procedures for fortification (e.g., Quality Assurance (QA) and Quality Control (QC) process) and adapting to a new fortification law and quality standards (Maestre et al., 2017). Lack of technical capacity to fortify and monitor the fortification level is another important constraint (Luthringer et al., 2015, Mark et al., 2019), particularly for small- and medium-scale enterprises.

As fortified and non-fortified oil products in Bangladesh are not well differentiated through labeling and certification (GAIN and icddr’b, 2017), competing non-fortified products in the market disincentivizes producers from investing in premix, fortification equipment and monitoring (Luthringer et al., 2015). While the demand for non-compliant products can be reduced through awareness raising among consumers, the *credence* nature of fortified products (Laviolette, 2018) makes it challenging as, without labeling, fortified and non-fortified products are often identical in their appearance, smell, and taste. Additionally, the positive effects of these micronutrients are not evident in the short term nor easily attributable to the food consumed over time. This is evident in the case of iodized salt in Bangladesh where the consumption of under-fortified salt persists despite decades of consumer awareness campaigns (OPM et al., 2020b). Moreover, better signaling of fortified products, such as through the development of certification schemes, has cost implications that could affect affordability.

In the context of the aforementioned market disincentives, government regulations and their enforcement crucially influence for-profit actors’ behaviors (Abdoulaye and Manus, 2018, Ebata et al., 2020). Where enforcement of requirements is weak, under-fortification, misleading labels or counterfeit products create unequal competition and provoke consumer suspicions. Many government agencies are under-funded and lack technical capacity and knowledge to appropriately enforce fortification regulations (Luthringer et al., 2015). Because LSFF programs require regulatory agencies to collect samples for laboratory testing and carry out quality and safety evaluations based on high-quality laboratories and effective surveillance mechanisms (Bennett et al., 1994, Hongoro and Kumaranayake, 2000, Raghavan et al., 2019), it is particularly challenging to monitor a large number of small-scale actors that are geographically sparse (Chadare et al., 2019).

Another challenge facing fortification programs is that regulatory responsibility is at times unclear amongst government agencies that are involved in ensuring adequate fortification (GAIN and PHC, 2018). This is partly because nutrition issues span different ministries in a government. Lack of coordination among government entities can lead to fragmented regulations for monitoring, quality control and inspection (Darnton-Hill et al., 2017). With unclear roles and responsibility assignment, confidence in the regulation is undermined and existing fortification regulations may be weakly or not at all enforced.

Corruption by enforcement agencies and political resistance from industry actors is another critical constraint to effective regulatory enforcement (Darnton-Hill et al., 2017, Luthringer et al., 2015). Indeed, the introduction of oil fortification law in Bangladesh was met with resistance by refineries as they anticipated increased regulatory oversight and control by the government (Kar, 2018). Regulators perceive political risk of enforcement and thereby are incentivized to be lenient in monitoring and reporting the violation of regulations (Luthringer et al., 2015). As a result, penalties might be kept too low to incentivize industry actors to consistently fortify and/or there might be inconsistent enforcement of the existing fortification standards (Luthringer et al., 2015).

In summary, the success of LSFF in LMICs will depend on the incentives shaping the behavior of private sector actors. These in turn are affected by market factors, such as competition between products, and the cost of production, as well as the regulatory and policy environment. In order to understand the relevance of these drivers of private sector compliance and how they interact to shape LSFF outcomes, we address the following research questions in the context of Bangladesh:

What demand and supply-side factors influence key decisions by actors in the edible oil value chains that affect fortification outcomes?
How do these factors enable or undermine the uptake of fortified foods, particularly among low-income groups?

## 3 Methods

### 3.1 Data collection strategies

We employed qualitative research methods to collect data regarding value chain dynamics in the edible oil sector in Bangladesh. Primary data was collected via semi-structured interviews and focus group discussions (FGDs) with value chain actors, consumers of edible oil, and key informants outside the value chains that have relevant expertise or knowledge about factors affecting fortification decisions. In total, we interviewed 4 refineries, 6 packers, 6 wholesalers, 7 retailers, one representative from the packers’ association, one premix supplier, and 5 key informants (Nutrition International, Scaling Up Nutrition (SUN) Business Network, GAIN Bangladesh, MoInd, and Bangladesh Small and Cottage industries Corporation (BSCIC)) and conducted 5 FGDs with consumers (Table 1).

**Table 1:** Geographical distribution of interviews and FDGs per stakeholder group.

Region Actor*	Chittagong	Dhaka	Narayanganj	Gazipur	Natore	Rangpur	Sylhet
Premix suppliers (N = 1)	0	1	0	0	0	0	0
Packers (N = 38)	1	1	1	1	0	0	2
Refineries (N = 28)	0	4	0	0	0	0	0
Wholesalers	2	1	0	0	2	1	0
Retailers	3	1	0	0	1	2	0
Consumer FGD	2	1	0	0	1	1	0
Key informants	0	5	0	0	0	0	0

*: N shows the total number of premix suppliers, refineries and packers based on our scoping study. There are 28 refineries across a total of 25 enterprise groups. We are unable to identify how many businesses exist in other stakeholder groups.

Stratified purposeful sampling (Patton, 2002) was used to identify value chain actors for interview, seeking to consult those that were considered ‘typical’ of their position in the value chain, while also seeking variation particularly in terms of business size and geography. As there was difficulty in reaching some market actors, notably oil refineries, there was also a degree of opportunistic sampling, taking advantage of circumstances that enabled access, including personal connections and snowballing approach. While this approach may have resulted in some participation bias, it allowed us to obtain insights from across the value chain, and from a wider range of respondents^6^. Moreover, although the number of refineries interviewed (4) is small, it represents a reasonable proportion of the total population (25 enterprise groups according to MoInd).

FGD participants were selected so that a diversity of opinions was captured across income levels and geographical areas. In addition, there was an effort to ensure that FGDs were gender balanced to capture any gendered patterns in consumer awareness and behavior. However, women were under-represented in FGDs in Chittagong and Dhaka, due to cultural barriers. While gender-specific factors may not be adequately captured, we did not find important gendered differences in the other FGDs.

As fortified oil consumption, distribution and production differed across country (OPM et al, 2020a), we purposively selected stakeholders in different parts of Bangladesh (Table 1).

We collected data in Natore, Rangpur, Narayanganj, Dhaka, Gazipur, Chittagong and Sylhet. These districts were selected based on the locations of oil facilities, as well as the socio-economic characteristics of the consumers in different parts of Bangladesh. For example, we selected Natore and Rangpur districts in the north because consumers tend to be poorer compared to the rest of the country.

We were unable to record interviews due to existing tensions related to policy reforms. In order to retain detailed information from the interviews, the field team extracted relevant information in a pre-defined template soon after the interviews. Studies such as Rutakumwa et al. (2019) endorse such an approach for interviews conducted in sensitive social settings. In adherence to the ethical standards and requirements of this research^7^, the responses that each of these study participants provided are anonymized, and/or aggregate findings are presented.

### 3.2 Data analysis

Our analysis is based on three sources of information: (1) desk-based data collection regarding the structure of the value chains; (2) primary data collected through key informant interviews, stakeholder interviews and FGDs; and (3) theoretical and empirical evidence from the literature. We coded interview notes according to the conceptual framework by Maestre et al. (2017) and compared information from all three sources to triangulate it.

Based on Maestre et al. (2017), we analyzed five demand-side requirements – 1) nutrition awareness, 2) signaling, 3) availability, 4) affordability, and 5) acceptability – and five supply-side requirements – 1) value creation and capture, 2) fair distribution of incentives, 3) value chain coordination and governance, 4) risk and uncertainty management, and 5) appropriate institutional environment – for value chains to provide nutritious food for vulnerable people. We apply the framework in order to identify demand- and supply-side factors that influence edible oil producers and shape fortification outcomes. While Maestre et al. (2017) is not exclusively focused on fortified foods, to the best of our knowledge, this is the only existing framework that helps us analyze the opportunities and challenges faced by private sector actors post-farm gate in delivering nutritious foods, unlike those focused on the farm-level production of such foods (Girard et al., 2012, Masset et al., 2012). Therefore, we adopted this framework for our analysis.

## 4 Results

### 4.1 Demand-side factors

#### 4.1.1 Availability and affordability

As found by GAIN and icddr’b (2017), our data show that both bulk and bottled oil products were widely available in all areas where we conducted fieldwork. Across the study sites, the difference between the cheapest bottled oil option and cheapest bulk oil option was between 3 and 35 BDT (Table 2). Low-income consumers and some of the middle-income consumers in Rangpur and Muhammadpur expressed preference for bulk oil over bottled oil because of the price difference. Generally, high-income consumers buy more bottled oil. FGDs in Chittagong indicate that all of the low-income consumers purchased bulk oil while all middle-income consumers purchased bottled oil.

**Table 2:** Oil prices paid by consumers across study sites (BDT per liter).

Location	Consumer characteristics	Bottled	Bulk		
		Soybean	Soybean	Super palm	Palm
Dhaka	Middle-income	95–102	90	85	NA
Natore	Rural low-income	95–105	85	65	60
Chittagong	Rural middle-income	88–110	NA*	NA*	NA*
Chittagong	Rural low-income	88–110	85–90	85–90	85–90
Rangpur	Middle-income	95–105	83–85	65	NA

*Middle-income consumers in Chittagong said that they do not purchase bulk oil.

However, affordability is not the only deciding factor behind consumer choices. Even low-income consumers purchase bottled oil during festivals and celebratory occasions to treat their guests. In some middle-income communities (e.g., FGD in Dhaka), consumers indicated that they prefer bulk oil for daily use and bottled oil only for special occasions, despite higher purchasing power. In other words, consumers evaluated the balance between price and quality of bulk and bottled oil in terms of the perceived benefits. In cases where the benefits of bottled oil are considered worthwhile, consumers will purchase bottled oil.

While value chain actors report that product availability is largely driven by consumer demand, other factors also influence the brands and suppliers they work with. Retailer interviews reveal that they choose products to stock depending on which products are distributed to their area, product costs and relationship with sellers. This relationship is mostly with respect to packers – who may offer flexible payment terms (credit) as an incentive for retailers to stock their products. Also, some packers allow retailers to return damaged, unsold or expired products. Retailers can make more profit out of bulk oil than bottled oil because the retail price of bottled oil is fixed by the Maximum Retail Price (MRP). MRP is defined and inspected by the government for various products, including edible oil. Since retailers face this cap on their revenues from bottled oil, their profits depend on the wholesale prices they are able to negotiate with the producers, creating pressure for packers to keep prices low.

Particularly in rural markets, wholesalers are influential in deciding from which refineries bulk oil is sourced. Each wholesaler distributes to particular areas. In Natore and Rangpur, the wholesalers we interviewed were the only ones in the market (i.e., they have a monopoly), unlike in urban areas such as Dhaka and Chittagong. In the case of bottled oil, multiple distributors operate in a given area. Some wholesalers and retailers, often large-scale ones, can attract clientele by offering credit.

#### 4.1.2 Nutrition awareness and signaling

Generally, consumer awareness of vitamin A fortification in edible oil was low. Out of the five consumer FGDs, there was only one FGD where two out of six participants were aware of fortification in oil. Nevertheless, consumers perceived bottled oil to be higher quality than bulk oil. Consumers judged quality based on the color of the oil, “cleanliness”, the quality of cooking (i.e., how food felt hours after cooking) and viscosity, but not whether oil contained vitamin A.

Signaling of credence characteristics is currently weak. Although consumers are largely unaware of fortification, they expressed general distrust of information on product labels. In particular, perceived incidences of mislabeling were cited such as palm oil being sold as soy. Currently there is no clear labeling of bulk oil. As a result, consumers do not receive any signaling in relation to fortification status of oil. It also means that the origin of bulk oil is not traceable.

### 4.2 Supply-side factors

#### 4.2.1 Value creation, capture and distribution

While we were unable to retrieve exact cost figures from firms, we estimated profits from selling bottled or bulk oils for refineries based on interviews with chemists working in the edible oil industry as below (Table 3). Assuming that refineries appropriately fortify both bottled and bulk oils, and premix from BASF costs BDT 15,000 per kg, a refinery could make 34–38% further profit per liter of bottled oil and 57–75% more profit per liter of bulk oil if the oil is not fortified. While these figures should be taken with caution, Table 3 highlights the relatively higher benefits of under-fortifying bulk oil and challenge the view that fortification cost is negligible for producers (Chaudhry, 2018).

**Table 3:** Cost, revenue and profit for refineries and packers (BDT per liter).

Details	Refineries		Packers
	Bottled oil	Bulk Oil	Bottled oil
Revenue per liter of oil	BDT 90	BDT 75	BDT 85
Gross profit*	BDT 6 to 7	BDT 5 to 6	BDT 4 to 5
Operating (net) profit**	BDT 2 to 2.5	BDT 1 to 1.5	BDT 1.5 to 2
Operating profit for non-fortified oil	BDT 2.75 to 3.35	BDT 1.75 to 2.35	BDT 2.25 to 2.85
Additional profit for non-fortified oil (%)	34–38%	57–75%	43–50%

*Revenue minus cost of producing fortified oil.

** Gross profit minus operational and administration costs.

Packers face stiff competition in the bottled oil market, both against each other, and with respect to the large refineries. Refineries acknowledge that packers can penetrate markets that are often out of reach for refineries, particularly in rural areas. However, refineries are trying to advance into these markets, thereby increasing competition in places where the competition among packers is already fierce. This creates pressure for packers to keep their prices and therefore costs low. Moreover, as packers only undertake packaging and distribution activities, their costs and margins are sensitive to a limited set of activities. Although the oil that packers purchase from refineries should already be fortified, packers are required to test and where vitamin A content is below the legal requirement, they are expected to fortify the oil before packaging it.

Regarding premix sourcing, BASF only sells premix in large volumes of 5 or 6 L. While refineries can purchase the required quantity at once, packers turn to a sales agent who sells premix in smaller quantities, usually 0.5 L. As a result, packers turn to Grade B premix which is cheaper and allegedly less stable than BASF premix (see footnote 5). Due to the competitive pressures discussed above, the minimum purchase volume stipulated by BASF and the lack of sufficient knowledge amongst packers regarding the suitability of alternative premix sources, packers use this Grade B premix.

#### 4.2.2 Value chain coordination and managing risks of investing in fortification

We identified no mechanisms to offset or share the costs of investing in the supply of fortified oil. Regarding QA/QC, refineries have their own full-time chemists to check fortification level. Packers indicated that they contract external chemists or equipment to check fortification levels. These chemists work with multiple packers. Packers also noted challenges associated with the productivity of in-house laboratory facilities. For instance, one packer expressed that their current equipment is slow at detecting fortification levels. Some packers do not actively check the fortification level, as they say that bulk oil coming from refineries should be fortified properly.

Moreover, value chain coordination is ineffective in signaling which products are appropriately fortified or not. Most wholesalers and retailers accept that the products they receive are fortified “to some extent” but some express doubts about these claims.

#### 4.2.3 Institutional environment influencing value chain coordination and governance

Tensions exist between the government and the association of refineries, the Bangladesh Vegetable Oil Refinery Association (BVORA), with regards to the effort to mandate oil fortification in the country (Kar, 2018). The law mandating oil fortification was enacted in 2010 by MoInd with support from the Global Alliance for Improved Nutrition (GAIN) and UNICEF (GAIN, 2019). However, BVORA resisted this initiative, which resulted in a delay in implementation of the legislation until 2015 (Kar, 2018).

In terms of monitoring and enforcing the oil fortification law, BSTI within the MoInd is the nodal agency for regulatory enforcement and monitoring. According to key informant interviews, BSTI conduct inspections of both refineries and packers, and these are expected to take place twice a year, once at production level where sites are visited and samples collected, and once at retail level.

At production level, inspections include the conditions of the premises and equipment, capability of personnel, the adequacy of internal quality control measures and equipment, and sampling of products. At the retail and market level, BSTI collects samples of bottled edible oil and tests fortification quality. Where under-fortified bottled oil is detected at retail level, it is traced back to the producers, and can result in reputational damage, as well as more formal penalties. In the case of bulk oil, however, a lack of labelling means that inadequately fortified bulk oil found in the market cannot be traced to the refinery that produced it.

Where producers are found to be in violation of the regulation, they are issued a warning and asked to submit a letter explaining why they failed to meet the standard in that instance. They are expected to carry out corrective actions, and if not, their license can be cancelled by BSTI. Fines may also be applied: ranging from BDT 50,000 (USD 575) for the first offence to BDT 200,000 (USD 2,300) for subsequent offences, which may be accompanied by imprisonment for terms ranging from six months to five years. One key informant argued that the level of fine imposed is too low for the refineries to motivate compliance.

## 5 Policy implications

We now synthesize the findings presented above to highlight the key obstacles to successful fortification of edible oil in Bangladesh. We also draw generalized lessons for improving private sector compliance to LSFF regulations in LMICs.

The first challenge is the persistent demand for under-fortified food. Our data shows that even middle-income consumers with higher purchasing power commonly choose bulk oil, implying that the added benefits of bottled, i.e., fortified, oil do not warrant the higher price on a regular basis. Fortification status of food items is invisible to consumers (Laviolette, 2018). As a result, consumer awareness regarding nutritional value of fortified food is low. There is little incentive for producers to supply appropriately fortified products or for other market actors to be concerned with the fortification level of the product they distribute and sell. As consumers do not consider fortification status in purchasing decisions, there is also little incentive to improve information sharing or otherwise resolve this issue within the value chain.

The second challenge is reflected in disincentives for large-scale producers, i.e., refineries, to fortify despite their ability to do so. While Chaudhry (2018) suggests that the cost of fortification is negligible for large-scale producers, our analysis indicates that saving the cost of premix allows them to increase profit particularly for bulk products. Moreover, even if sub-standard oil is detected in the market, bulk oil cannot be traced to specific producers due to lack of labeling, meaning producers receive no sanction. The only penalty is through reputational damage if their bottled oil fails to meet legal fortification requirements. This explains why traceable products are better fortified than bulk items.

The third challenge is reflected in the economic constraints facing small-scale producers, i.e., packers, who supply edible oil in rural areas for marginal communities. Unlike refineries, packers undertake fewer activities through which they can generate a margin or where they can seek cost efficiencies. As they face substantial competition from other packers and refineries, packers’ margins are squeezed. Another source of pressure might have been from providing credit to gain a loyal customer base amongst retailers, constraining their cash flow, as described in Randall and Anjum (2014). These economic pressures incentivize packers to use low-quality premix. In addition, given the significant variation in price and quality of premix available through third-party vendors in the wholesale markets in Bangladesh, it is also difficult for packers to adequately assess quality and ensure they pay an appropriate price (Garrett et al., 2015).

The fourth challenge is inconsistent law enforcement. Effective enforcement is key to compliance, which requires consistent inspection and enforcement conducted as close to production sites as possible (Luthringer et al., 2015, van den Wijngaart et al., 2013). However, the current regulation requires both large- and small-scale producers to fortify, leading to confusion and increased costs by small-scale producers. Moreover, while BSTI inspects bottled oil, the fortification status of bulk oil in the market is currently not monitored. BSTI does inspect refineries at the production level, where problems with fortification should be identified. However, the persistence of under-fortified bulk oil suggests that these measures have been ineffective. Given that the introduction of LSFF was strongly resisted by BVORA (Kar, 2018), BSTI may also be subject to political risks and pressure from the producer association (Luthringer et al., 2015).

To improve the fortification status of edible oil products, Indonesia (Jus’at and Soekirman, 2019) and Pakistan (Randall and Anjum, 2014) banned the retail sale of bulk oil. However, our analysis indicates that such a policy change would lead to several challenges. First, this does not effectively address the fortification of bulk oil purchased and packaged by small-scale producers. As large-scale producers supply them with bulk oil, one could argue that the responsibility to fortify bulk oil is of refineries. While small-scale producers could be required to bear the full responsibility for fortification, they will face challenges to keep cost low, procure adequate premix and to test the fortification status of bulk oil they procure. Secondly, it could jeopardize availability or affordability of edible oil for marginalized communities. Therefore, such a policy instrument would likely be unpopular with consumers, who prioritize affordability.

Our findings suggest that a preferred alternative would be to better enforce fortification of bulk oil at refinery level. As bulk oil is exclusively produced by them and is the main input for the bottled oil produced by packers, it would reaffirm refineries’ primary role and responsibility for fortification (Bishai and Nalubola, 2002; Luthringer et al., 2015) and remove this responsibility from packers who are less able to adequately fortify. It would also enable the government to focus enforcement efforts on the smaller number of large-scale producers and makes it unnecessary to improve consumer awareness.

Enforcement can focus on low-cost strategies such as correlating the amount of premix used by producers to the amount of fortified food said to be produced (GAIN, & PHC, , 2018, Luthringer et al., 2015, Mbuya et al., 2020). Such data is currently submitted to BSTI by refineries, but is not being verified. Also, all fortified products, including bulk items, should be traceable through appropriate labelling in order to act as a final validation/check on fortification. Penalty needs to be sufficiently impactful where infringements are detected.

Better enforcement may still be affected by political risks, which are likely to require new approaches. It may include stronger information and communication efforts involving a consistent dialogue between large-scale actors and regulatory authorities. Dialogue can support refineries to become more comfortable with the requirements, to gain trust and confidence in the regulatory process, and to develop a more positive attitude concerning the importance of fortified bulk oil (Luthringer et al., 2015, Mbuya et al., 2020, Soekirman et al., 2012). These measures would require sufficient regulatory agency resources and capacity to ensure they are effective.

Together, these efforts would significantly improve fortification outcomes with respect to bulk items. If these improvements were to be achieved, they would in turn improve the likelihood that the bulk items purchased and bottled by small-scale producers would be already adequately fortified. In addition, small-scale producers would benefit from support to perform adequate quality control on the oil they purchase and to procure suitable quality premix where necessary. For example, the government and its partners could facilitate the development of central or collective purchasing, storage and on-sale, as well as common testing to optimize premix acquisition and quality control (Mkambula et al., 2020).

## 6 Conclusions

In this paper, we analyze mechanisms in which for-profit actors comply with large-scale food fortification programs aiming at improving micronutrient deficiency based on a case study of the edible oil sector in Bangladesh. While our research is timely for Bangladesh where the mandatory oil fortification regulation is currently under review, it also provides other governments in low- and middle-income contexts who aim to improve nutritional outcomes of marginalized population. We elicit factors influencing value chain actors’ decisions whether or not to comply with the fortification law, and by how much. We situated our analysis in the framework by Maestre et al. (2017). Their framework is designed to assess the effectiveness of agri-food value chains at improving the nutrition intake of vulnerable groups, and to the best of our knowledge, our study is the first to apply this framework systematically to an empirical context.

Our analysis identified several key bottlenecks for LSFF programs in LMICs. First, under-fortified products are popular in the market, disincentivizing value chain actors from supplying fortified products. Second, the lack of traceability for bulk items discourages large-scale producers to comply with the regulation. Third, small-scale producers face cost pressures that prevent them from adequately fortifying oil products. Lastly, law enforcement is currently insufficient to prevent large-scale producers from supplying under-fortified bulk items in the market. Given these constraints, we recommend that policy makers strengthen the control of bulk item fortification through stronger engagement with large-scale producers. This approach will reaffirm the responsibility of large-scale producers to fortify oil thoroughly and thereby allow the government to control a smaller number of large-scale producers. This will ensure that marginalized consumers remain able to access affordable oil products without improving consumer awareness.

## CRediT authorship contribution statement

**Ayako Ebata:** Conceptualization, Data curation, Formal analysis, Investigation, Methodology, Validation, Writing - original draft, Writing - review & editing. **Jodie Thorpe:** Conceptualization, Data curation, Formal analysis, Investigation, Methodology, Project administration, Validation, Writing - review & editing. **Ainee Islam:** Data curation, Investigation, Methodology, Project administration, Supervision, Writing - review & editing. **Sabiha Sultana:** Conceptualization, Funding acquisition, Methodology, Writing - review & editing. **Mduduzi N.N. Mbuya:** Conceptualization, Funding acquisition, Methodology, Project administration, Supervision, Validation, Writing - review & editing.

## Declaration of Competing Interest

The authors declare that they have no known competing financial interests or personal relationships that could have appeared to influence the work reported in this paper.

## Footnotes

1 Artisanal production of edible oil in Bangladesh such as mustard oil only accounts for less than 10% of vegetable oil available in Bangladesh.

2 The majority of imported refined oil consists of sunflower oil, which was not included in this study.

3 Packers and refineries do not have contractual arrangements that specify the quantity of oil purchase, timing, etc.

4 An informant indicated that, unlike Grade A premix, Grade B premix may deteriorate after a period of exposure to sunlight as it lacks antioxidant. However, we were unable to confirm this independently.

5 An informant indicated that, unlike Grade A premix, Grade B premix may deteriorate after a period of exposure to sunlight as it lacks antioxidant. However, we were unable to confirm this independently.

6 At the time of the fieldwork, mandatory oil fortification law was under revision, which led to significant tension between MoInd and oil refineries. As a result, our initial attempt to interview refineries was rejected without employing snowballing approach or recruitment through personal contacts.

7 The study received ethical approval from the Institutional Review Board (IRB) of the Institute of Health Economics at the University of Dhaka, which was issued by the IRB on 19 August 2019.
